# Epidemiology and outcome of sepsis syndromes in Italian ICUs: a regional multicenter observational cohort

**DOI:** 10.1186/cc11004

**Published:** 2012-03-20

**Authors:** L Laudari, Y Sakr, C Elia, L Mascia, B Barberis, S Cardellino, S Livigni, G Fiore, C Filippini, VM Ranieri

**Affiliations:** 1 San Giovanni Battista-Molinette Hospital, University of Torino, Turin, Italy; 2 Friedrich Schiller University Hospital, Jena, Germany; 3 Ospedale degli Infermi, Revoli, Italy; 4 Ospedale Cardinal Massaia, Asti, Italy; 5 Ospedale Giovanni Bosco, Turin, Italy; 6 Ospedale Santa Croce, Moncalieri, Italy

## Introduction

We assessed the epidemiology of sepsis syndromes in patients admitted to ICUs of the Piedmont region in northern Italy and investigated the impact of sepsis on ICU mortality in these patients.

## Methods

In this prospective, multicenter, observational study, all 3,902 patients (mean age ± SD: 64.3 ± 15.7 years, 63.5% male) admitted to one of 24 medical or surgical ICUs between 3 April and 29 September 2006 were included.

## Results

Four hundred and forty-six of the patients had sepsis, including 160 patients with severe sepsis (4.1%) and 145 patients (3.7%) with septic shock. ICU mortality was 20% (*n *= 780) and median ICU length of stay was 3 (1 to 9) days. ICU mortality was higher (41.3 vs. 17.2%, *P *< 0.001) and the median ICU LOS longer (15 (7 to 26) vs. 2 (1 to 7), *P *< 0.001) in patients with sepsis than in those without sepsis. The mortality rate increased with the severity of sepsis (sepsis without organ failure, severe sepsis, and septic shock: 19.9, 44.4, and 58.6%, respectively). ICU-acquired sepsis was associated with higher ICU mortality rates than sepsis occurring within 48 hours of ICU admission (49.8 vs. 33.0%, *P *< 0.001). In multivariate logistic regression analysis, the occurrence of severe sepsis (OR, 1.70 (1.06 to 2.72); *P *= 0.026) and septic shock (OR, 2.25 (1.49 to 3.49); *P *< 0.001) were independently associated with an increased risk of ICU death.

## Conclusion

In this large multicenter cohort, severe sepsis and septic shock were independently associated with an increased risk of death. Our data underscore the regional variability in the epidemiology and outcome of sepsis syndromes and may be useful for resource allocation.

